# Functional weight-bearing mobilization after Achilles tendon rupture enhances early healing response: a single-blinded randomized controlled trial

**DOI:** 10.1007/s00167-016-4270-3

**Published:** 2016-08-18

**Authors:** Kars P. Valkering, Susanna Aufwerber, Francesco Ranuccio, Enricomaria Lunini, Gunnar Edman, Paul W. Ackermann

**Affiliations:** 1 Orthopaedics Department, Orthopedium, Delft, The Netherlands; 2 Physiotherapy Department, Karolinska University Hospital, Stockholm, Sweden; 3 Orthopaedics Department, School of Medicine, Magna Graecia University, Catanzaro, Italy; 4 Orthopaedics Department, Azienda University Hospital Parma, Parma, Italy; 5 Department of Clinical Neuroscience, Karolinska Institutet, Stockholm, Sweden; 6 Integrative Orthopedic Laboratory, Department of Molecular Medicine and Surgery, Karolinska Institutet, Stockholm, Sweden; 7 Orthopedic Department, Karolinska University Hospital, 171 76 Stockholm, Sweden

**Keywords:** Achilles tendon, Metabolism, Revalidation, Collagen, Rupture, Metabolite, Microdialysis

## Abstract

**Purpose:**

Functional weight-bearing mobilization may improve repair of Achilles tendon rupture (ATR), but the underlying mechanisms and outcome were unknown. We hypothesized that functional weight-bearing mobilization by means of increased metabolism could improve both early and long-term healing.

**Methods:**

In this prospective randomized controlled trial, patients with acute ATR were randomized to either direct post-operative functional weight-bearing mobilization (*n* = 27) in an orthosis or to non-weight-bearing (*n* = 29) plaster cast immobilization. During the first two post-operative weeks, 15°–30° of plantar flexion was allowed and encouraged in the functional weight-bearing mobilization group. At 2 weeks, patients in the non-weight-bearing cast immobilization group received a stiff orthosis, while the functional weight-bearing mobilization group continued with increased range of motion. At 6 weeks, all patients discontinued immobilization. At 2 weeks, healing metabolites and markers of procollagen type I (PINP) and III (PIIINP) were examined using microdialysis. At 6 and 12 months, functional outcome using heel-rise test was assessed.

**Results:**

Healing tendons of both groups exhibited increased levels of metabolites *glutamate, lactate*, *pyruvate,* and of *PIIINP* (all *p* < 0.05). Patients in functional weight-bearing mobilization group demonstrated significantly higher concentrations of glutamate compared to the non-weight-bearing cast immobilization group (*p* = 0.045).The upregulated glutamate levels were significantly correlated with the concentrations of PINP (*r* = 0.5, *p* = 0.002) as well as with improved functional outcome at 6 months (*r* = 0.4; *p* = 0.014). Heel-rise tests at 6 and 12 months did not display any differences between the two groups.

**Conclusions:**

Functional weight-bearing mobilization enhanced the early healing response of ATR. In addition, early ankle range of motion was improved without the risk of Achilles tendon elongation and without altering long-term functional outcome. The relationship between functional weight-bearing mobilization-induced upregulation of glutamate and enhanced healing suggests novel opportunities to optimize post-operative rehabilitation.

**Electronic supplementary material:**

The online version of this article (doi:10.1007/s00167-016-4270-3) contains supplementary material, which is available to authorized users.

## Introduction

In acute Achilles tendon rupture (ATR), there is still lack of consensus regarding the best post-operative rehabilitation. There is a controversy regarding range of motion and the amount of weight-bearing or immobilization. More recently, studies have reported a positive effect of early, functional weight-bearing (FWB) rehabilitation in both conservative and operative treatment [8, 15, 22, 32]. However, the underlying mechanisms behind early mobilization, and whether direct post-operative FWB can affect early ATR healing, have not been clarified.

Research suggests that immobilization causes a lower metabolic activity and collagen production, which can be upregulated with early functional mobilization [9, 28]. Assessment of metabolism and healing progression after ATR has recently been made possible by the development of in vivo microdialysis technique. Thereby, several metabolites, e.g. glutamate, essential for energy provision and cell proliferation, as well as markers of callus production can be quantified [1, 13].

The effects of direct post-operative FWB on healing metabolite levels and callus production during ATR repair in vivo as well as long-term functional outcome were unknown and needed to be better delineated. We hypothesized that ATR repair involves a differential metabolite upregulation responsive to FWB, which would lead to increased callus production and improved patient functional outcome. A randomized controlled trial (RCT) to determine the relationship between functional weight-bearing and metabolite levels was performed.

## Materials and methods

This study was designed and reported according to the CONSORT (Consolidated Standards of Reporting Trials) guidelines [23]. This study was conducted at the Karolinska University Hospital, Stockholm, Sweden. All participants received oral and written information about purpose and procedures of the study and provided written informed consent prior to surgery. The trial was registered under the reference number (www.clinicaltrials.gov; trial number NCT02318472). This was a single-centre, single-blinded, controlled, parallel-group study using block randomization.

## Participants, eligibility criteria and randomization

Patients between 18 and 75 years of age with a unilateral ruptured Achilles tendon were included, provided they could give informed consent and if surgery was performed within 1 week after the injury. The exclusion criteria were: current anticoagulation treatment (including high-dose acetylsalicylic acid), known kidney failure, heart failure with pitting oedema, thrombophlebitis, thromboembolic event during the previous 3 months, known malignancy, haemophilia, pregnancy, other surgery during the previous month, inability to follow instructions and planned follow-up at another hospital.

Post-operatively a non-stratified block randomization was used by the senior author assigning the patient to either direct post-operative functional mobilization with full weight-bearing group (FWB) or immobilization and non-weight-bearing group (IMM). A computer program was used to generate random numbers in permuted blocks of six, and patients were allocated in a ratio 2:1. The 2:1 allocation ratio was chosen by recommendations from the ethical committee since our hypothesis is that the mobilized group will perform better, and data were available of fifteen patients from an immobilized group from a previous study [13].

### Patient characteristics

**Fig. 1:**
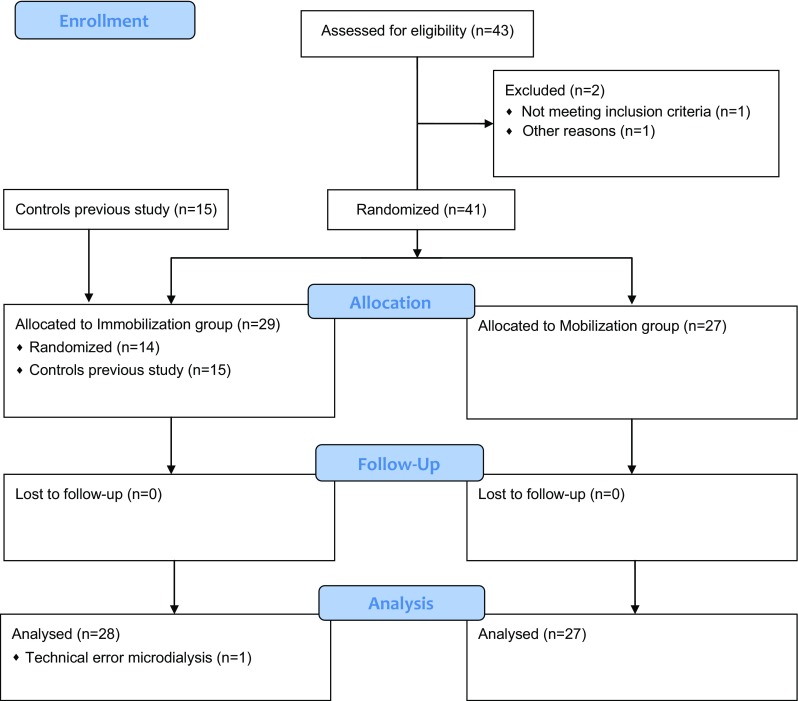
CONSORT study flow diagram

Between November 2013 and November 2014, a total of 43 patients were assessed for eligibility in this study (Fig. 1). Two patients were excluded because in one patient, a muscle rupture rather than an ATR was diagnosed and the other patient was excluded because thromboprophylaxis was prescribed pre-operatively. The remaining 41 patients were randomized and allocated to the study groups. Combined with 15 immobilized patients from our previous study, the total amount of patients included in this study was 56. The two patient cohorts were compared, and no significant differences were observed in the microdialysis results (*p* > 0.05).

In total, there were 50 men and 6 women with a mean age of 40.1 (SD 7.5) years (range 25–58). A significant longer time interval between injury and surgery was seen in FWB group compared to the IMM group (Table 1).

**Table 1:** Patient characteristics of the IMM and FWB groups

Group	FWB (*n* = 27) Number (%)		IMM (*n* = 29) Number (%)		*P*
Gender					n.s.
Male	24 (89)		26 (90)		
Female	3 (11)		3 (10)		
Nicotin					n.s.
Non	25 (93)		27 (93)		
Dipping tobacco	2 (7)		0		
Smoking	0		2 (7)		
ATR side					n.s.
Right	10 (37)		15 (52)		
Left	17 (63)		14 (48)		
	Mean (SD)		Mean (SD)		
Age (years)	40.8 (6.0)		39.5 (8.7)		n.s.
Height (cm)	180 (8)		180 (9)		n.s.
Weight (kg)	80.6 (11.5)		83.1 (13.9)		n.s.
BMI	24.9 (2.0)		25.6 (3.1)		n.s.
Time to surgery (hh:mm)	78:15 (42:24)		57:27 (22:12)		**0.029**
Time in surgery (hh:mm)	0:46 (0:18)		0:39 (0:11)		n.s.

Bold values represent significance at *P* < 0.05

*ATR* Achilles tendon rupture, *BMI* Body mass index, *FWB* Full weight-bearing and functional mobilization, *IMM* Immobilization and non-weight-bearing, *n.s.* non-significant

### Achilles tendon suturing technique and perioperative care

#### Surgical procedure

The surgical procedure was performed on an outpatient basis using local anaesthesia and a surgical technique as earlier described [12]. The surgical procedures were performed by specialists and resident orthopaedic surgeons from one university hospital. The surgical techniques were standardized for suturing technique and suture type.

#### Mobilized and full weight-bearing group (FWB)

Functional mobilization was initiated directly post-operative by the physiotherapist. An orthosis (VACO^®^ped, OPED Gmbh, Germany) with adjustable range of motion of the ankle was used. During the first two post-operative weeks, 15°–30° of plantar flexion was allowed. At 2 weeks post-operatively, this was increased to 5°–30° of plantar flexion for the remaining 4 weeks. Full weight-bearing with crutches and range of motion exercises were allowed after application of the orthosis. One hour daily non-weight-bearing range of motion exercises without the orthosis was recommended.

#### Immobilized and non-weight-bearing group (IMM)

The IMM group received a conventional non-weight-bearing below-knee plaster cast with the ankle in 30° of equinus position. At 2 weeks post-operatively, the cast was replaced by a removable walker (Aircast^®^ Standard walking brace, DJO International, Surrey, UK) with three heel wedges for the remaining 4 weeks of immobilization. Every consecutive week, a heel wedge was removed. Full weight-bearing with crutches was allowed after application of the walker. One hour daily non-weight-bearing range of motion exercises without the walker was recommended.

### Follow-up evaluations

#### Microdialysis

To assess tendon healing, microdialysis followed by metabolite quantification was performed as described earlier [13, 20]. Microdialysis was conducted 2 weeks post-operative, when the primary plaster cast or orthosis was removed in the outpatient clinic. The microdialysis examiner was blinded to the intervention.

#### Metabolite analysis

The metabolites analysed in the microdialysis dialysate were *glutamate, glucose, glycerol, lactate, pyruvate* and *lactate/pyruvate* ratio using the ISCUS Clinical Microdialysis Analyzer (CMA Microdialysis AB, Solna, Sweden) as described earlier [13].

#### Measurement of procollagen type I and III and of total protein content

To assess markers of callus production quantifications of procollagen I N-Terminal propeptide (PINP), procollagen III N-Terminal propeptide (PIIINP) and of protein content were performed in the microdialysis dialysate [4].

#### Range of motion

Bilateral ankle dorsal flexion was assessed 2 weeks post-operative. The physiotherapist calculated an average of three recordings with a hand-held goniometer of the maximum amount of dorsiflexion of the ankle.

#### Functional outcome

Muscular endurance testing was conducted at 6 and 12 months post-operatively on both limbs by the physiotherapist. MuscleLab^®^ (Ergotest Technology, Oslo, Norway) measurement system was used for the evaluations and was performed as previously described in the literature [30, 31]. The Limb Symmetry Index (LSI = (injured/uninjured) × 100) was calculated for heel-rise height, repetitions and total work [31].

Ethical approval, Dnr 2013/1791-31/3, was obtained from the Regional Ethical Review Committee in Stockholm, Sweden.

### Statistical analysis

The sample size was calculated on a difference of the glutamate metabolite of 12 µM between the two groups. For this power analysis, we used a glutamate standard difference of 15 µM resulting from a previous study [13]. It was determined that a sample size of 25 patients per group would be necessary to detect the glutamate difference with 80 % power when alpha was set equal to 5 %. For the functional outcome, the sample size was calculated on a difference of total work of heel rise of 500 J. It was determined that a sample size of 23 patients per group would be necessary to detect the glutamate difference with 80 % power when alpha was set equal to 5 %. Anticipating that we would lose 10 % of participants enrolled, we had planned to enrol 27 patients in each group.

Since the microdialysis data showed a skewed distribution, the non-parametric Mann–Whitney *U* test was chosen for the assessment of metabolite and collagen and protein results. A non-parametric Levene’s test was used to verify the equality of variances in the samples (homogeneity of variance) (*p* > 0.05). Differences between the sutured ATR vs. the contralateral intact Achilles tendon were calculated with Wilcoxon matched pairs test. Pearson’s and Spearman’s correlations were calculated between the concentrations of the metabolites (normal distribution of glucose, pyruvate, and glutamate data in both study groups and lactate in the FWB group) and markers of callus production (normal distribution of data). The significance level in all analyses was 5 per cent (two-tailed). All statistical analyses were conducted using SPSS version 22 (IBM corporation, Armonk, New York, USA).

## Results

### Metabolite concentrations

At 2 weeks post-operatively, the healing Achilles tendons (AT) of both groups exhibited elevated metabolite concentrations of *glutamate*, *lactate* and *pyruvate*, as compared to the intact contralateral AT (Tables 2, 3). The levels of *glucose, glycerol* and the *lactate/pyruvate* ratio were, however, not significantly altered between the healing and intact AT in both the FWB and IMM groups (Tables 2, 3).

**Table 2:** Metabolite levels in the healing and intact contralateral tendons of the FWB group

Mobilized group	Sutured ATR			Intact contralateral AT			z	*P*	% Change
	*n*	*M*	SD	*n*	*M*	SD			
Glutamate (µM)	24	93.5	35.9	25	28.0	24.9	−4.1	**0.000**	234
Lactate (mM)	27	1.6	0.5	25	0.8	0.39	−4.1	**0.000**	99
Pyruvate (µM)	27	91.2	27.6	23	53.4	20.7	−3.9	**0.000**	71
Glucose (mM)	27	2.9	0.7	25	2.6	0.8	−1.1	n.s.	10
*L*/*P* × 1000	27	17.6	4.5	23	16.9	9.0	−0.9	n.s.	4
Glycerol (µM)	26	118	118	23	153	162	−1.1	n.s.	−22

Bold values represent significance at *P* < 0.05

*AT* Achilles tendon, *ATR* Achilles tendon rupture, *FWB* Full weight-bearing and functional mobilization, *n.s.* non-significant

**Table 3:** Metabolite levels in the healing and intact contralateral tendons of the IMM group

Immobilized group	Sutured ATR			Intact contralateral AT			z	*P*	% Change
	*n*	*M*	SD	*n*	*M*	SD			
Glutamate (µM)	26	73.8	34.9	27	22.7	14.6	−4.2	**0.000**	225
Lactate (mM)	28	1.5	0.9	25	0.7	0.3	−4.3	**0.000**	98
Pyruvate (µM)	28	80.6	27.5	25	48.9	30.8	−3.2	**0.001**	65
Glucose (mM)	28	2.7	0.8	27	2.5	0.9	−1.3	n.s.	11
Glycerol (µM)	28	135	218	27	162	177	−1.7	n.s.	−17
*L*/*P* × 1000	28	19.1	10.9	23	25.3	23.9	−1.3	n.s.	−24

Bold values represent significance at *P* < 0.05

*AT* Achilles tendon, *ATR* Achilles tendon rupture, *IMM* Immobilization and non-weight-bearing, *n.s.* non-significant

**Fig. 2:**
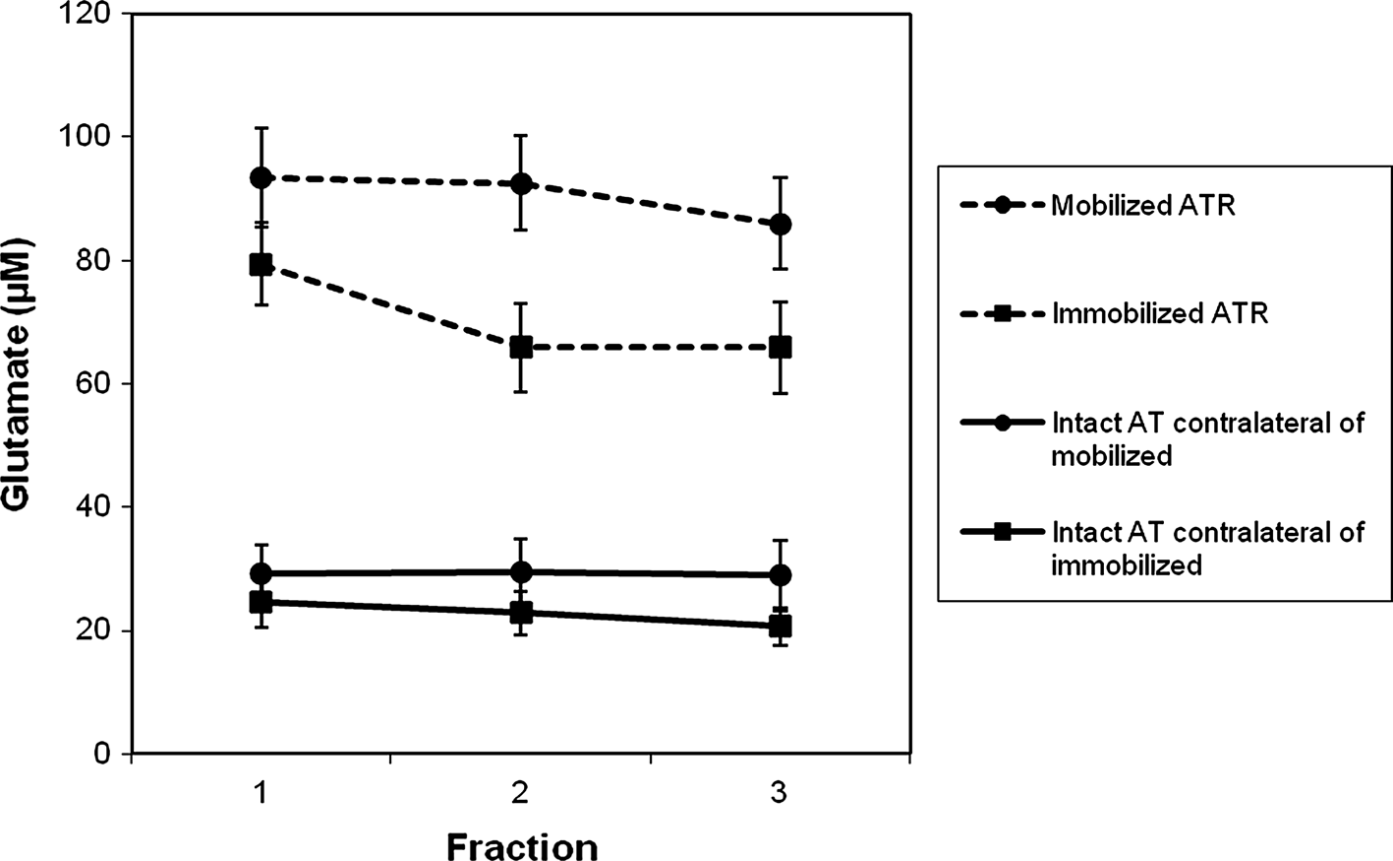
Glutamate peritendinous Achilles tendon microdialysis concentrations. Mean values and standard error to the mean are displayed

Comparison between the groups demonstrated that the FWB group exhibited significantly higher glutamate levels as compared to the IMM group (Table 4; Fig. 2). The other metabolite concentrations did not display any significant differences between the two groups (Table 4). Moreover, the metabolite concentrations in intact Achilles tendons did not demonstrate any significant differences between the groups (Table 5).

**Table 4:** Metabolite levels in the healing tendons comparing the FWB and IMM groups

Sutured ATR	Mobilized			Immobilized			z	*P*	% Change
	*n*	*M*	SD	*n*	*M*	SD			
Glutamate (µM)	24	93.5	35.9	26	73.8	34.9	−2.0	**0.045**	27
Pyruvate (µM)	27	91.2	27.6	28	80.6	27.5	−1.3	n.s.	13
Lactate (mM)	27	1.6	0.5	28	1.5	0.9	−1.4	n.s.	7
Glucose (mM)	27	2.9	0.7	28	2.7	0.8	−1.0	n.s.	6
*L*/*P* x 1000	27	17.6	4.5	28	19.1	10.9	−0.2	n.s.	−8
Glycerol (µM)	26	118	118	28	136	219	−0.7	n.s.	−12

Bold values represent significance at *P* < 0.05

*ATR* Achilles tendon rupture, *FWB* Full weight-bearing and functional mobilization, *IMM* Immobilization and non-weight-bearing, *n.s.* non-significant

**Table 5:** Metabolite levels in the intact tendons comparing the FWB and IMM groups

Intact AT	Mobilized group			Immobilized group			z	*P*	%Change
	*n*	*M*	SD	*n*	*M*	SD			
Glutamate (µM)	25	28	24.9	27	22.7	14.6	−0.3	n.s.	23
Pyruvate (µM)	23	53.4	20.7	25	48.9	30.8	−0.8	n.s.	9
Glucose (mM)	25	2.6	0.8	27	2.5	0.9	−0.9	n.s.	6
Lactate (mM)	25	0.8	0.4	25	0.7	0.3	−0.1	n.s.	5
Glycerol (µM)	23	153	162	27	162	177	−0.2	n.s.	−6
*L*/*P* x 1000	23	16.9	9.0	23	25.3	23.9	−1.7	n.s.	−33

*AT* Achilles tendon, *FWB* Full weight-bearing and functional mobilization, *IMM* Immobilization and non-weight-bearing, *n.s.* non-significant

### Markers of tendon callus production

At 2 weeks, the healing AT, compared to the contralateral intact AT, exhibited significantly higher concentrations of *PIIINP*, in both the IMM (*p* = 0.006) and FWB (*p* = 0.028) groups, while the levels of *PINP and protein* were not significantly altered. Comparison between the FWB and IMM groups exhibited no significant differences in the markers of tendon callus production.

### Markers of tendon callus production correlated to metabolites

In the FWB group, the concentrations of PINP in the healing Achilles tendons correlated significantly with the levels of glutamate (*r* = 0.6, *p* = 0.005), but not in the IMM group.

The concentration of PINP in the healing AT of the FWB group also exhibited a trend to correlate with the levels of lactate (*r* = 0.4, *p* = 0.051) and lactate/pyruvate ratio (*r* = 0.4, *p* = 0.070), but not in the IMM group.

### Functional assessments

At 2 weeks post-operatively, both the IMM and FWB groups exhibited significantly less dorsal flexion on the injured compared to uninjured side (*p* < 0.001). Additionally, total work (J) and heel-rise height (cm) exhibited significant differences between the injured and uninjured side at 6 months (*p* < 0.001), while at 12 months, total work was significantly different between the injured and uninjured side of the FWB (*p* = 0.001), but not of the IMM group. At 1 year post-operatively, LSI of total work was slightly higher in the IMM group (87.5 ± 25.8 J) compared to FWB group (74.9 ± 19.6 J); however, the difference was not significant.

When comparing functional outcome of the injured side, the FWB group demonstrated better outcome only at the early time point. Thus, ankle dorsiflexion at 2 weeks of the injured side of the FWB group was significantly better (*p* < 0.000) when compared to the IMM group. Furthermore, the LSI for dorsal flexion was significantly better for FWB groups compared to the IMM group. At 6 and 12 months, both groups demonstrated similar outcome in the heel-rise tests.

### Metabolites correlated to functional outcome

The post-operative glutamate concentrations in the healing Achilles tendons were significantly correlated to the active heel-rise height at 6 month post-operatively (*r* = 0.5; *p* = 0.014). At one year post-operatively, however, no significant correlation between these variables was detected.

### Complications

No harms or unintended effect was recorded in both groups due to the microdialysis procedure. One patient in the IMM group suffered from a traumatic rerupture after successfully completing the microdialysis and was not available for functional assessment at 6 and 12 months post-operative.

## Discussion

This study demonstrated that patients directed to functional weight-bearing mobilization compared to conventional plaster cast immobilization exhibited a further enhancement of the metabolic healing response after ATR repair. The elevated levels of the essential metabolite, i.e. glutamate, unravel one important molecular mechanism involved ATR healing, which is connected with increased collagen I synthesis and improved mid-term functional outcome. The data from this study could be used to improve outcome in ATR patients.

The FWB protocol applied in this study is to the best of our knowledge the first time that full weight-bearing combined with range of motion has been allowed direct post-operatively ATR and tested in a RCT study. The finding that the long-term heel-rise height did not differ between the two groups indicates that the FWB protocol did not elongate the Achilles tendons [31]. This conclusion is also supported from a recent roentgen stereophotogrammetric analysis on ATR healing that applied early tensional loading, which improved the mechanical properties without tendon elongation [27]. In addition, after non-operative dynamic treatment of ATR, immediate weight-bearing was also found to be a safe treatment modality with regard to functional outcome measures [5].

The novel results from this study pertained to that FWB increased the early healing response as observed by the 2 weeks improved ROM and upregulated the local concentrations of glutamate. Glutamate was the metabolite demonstrating the highest elevation during ATR repair, suggesting that glutamate is a substance with a more influential role in tendon healing, which is supported by earlier studies [24]. The observed increased glutamate levels in the FWB group thus suggest an improved healing process, supposedly in response to functional weight-bearing.

The observations that ATR repair entails a significant metabolic increase in glutamate, lactate and pyruvate corroborate the findings of a previous article [13] and further indicate that the upregulated substances are related to activate local metabolic pathways involved in the healing process of ATR.

The elevated glutamate concentrations in the FWB group observed at 2 weeks post-surgery indicate that the glutamatergic pathways exert effects in the early reparative/proliferative healing phase (ca. 1–6 weeks) [2]. Glutamate is known to be a chemotaxis-inducing factor for human neutrophils, and glutamate has demonstrated beneficial effects on both mucosal and epidermal wound healing [10, 14, 21].

Upregulated glutamate in the reparative phase presumably acts on adjacent cells, blood vessels and nerve fibres, where glutamate receptors have been identified in tendons [3, 29]. The glutamatergic actions in tendon repair may therefore be speculated to involve cell proliferation, angiogenesis and nerve ingrowth.

In tendon healing, nerve fibre and blood vessel ingrowth into the tendon proper, normally devoid of neurovascular supply, have been demonstrated essential to provide mediators to the repair site [2]. The upregulated glutamate levels may therefore reflect direct involvement in tendon repair relating to coordination of angiogenesis and nerve ingrowth. Glutamate is also involved in development, maintenance and healing of bone tissue [11, 16] and could therefore be seen as a metabotrophic regulatory substance of connective tissue in general.

The observed increase during healing of lactate and pyruvate in the repairing tendon may be related to intensify anaerobic and aerobic metabolism, respectively [25]. Lactate and pyruvate are known mediators in the early wound healing process. While the repairing tendons exhibited significant elevations of pyruvate and lactate, the early mobilization treatment protocol versus immobilization only produced small elevations of lactate and pyruvate, which were not significant. These findings indicate that the early mobilization protocol lacks the potential to significantly improve the aerobic or anaerobic metabolism at 2 weeks post-operatively.

The non-altered concentrations of glucose and glycerol during tendon healing suggest that these metabolites are not of major importance for the early repair process, at least not during the reparative phase.

Whether upregulated glutamate levels by the direct weight-bearing combined with the functional mobilization protocol can be attributed to mechanical loading, increased blood flow or nerve activity is not fully clear from this study. Bone formation has been demonstrated to be mechanical load-induced via glutamatergic signaling [7]. The finding that glutamate levels also were increased in the Achilles tendon of the un-loaded plaster cast patients indicates that additional pathways to loading were involved. Earlier studies have demonstrated increased peritendinous blood flow with muscle contraction in healthy individuals [6, 17, 18]. The functional mobilization protocol may, moreover, lead to increase neuronal activity as compared to plaster cast immobilization, which may contribute to improved glutamate levels as earlier demonstrated [2].

The non-significant differences in PINP, PIIINP and protein levels between the two groups indicate that the synthesis of proteins and of type I and III collagens at this time point of healing was not influenced by the functional weight-bearing protocol. Interestingly, however, was the finding that neither PINP nor protein levels were upregulated in the healing Achilles tendons, suggesting that the 2 weeks time point was too early to increase synthesis of proteins and of type I collagens. This conclusion is supported by experimental studies, demonstrating that the main upregulation of type I collagen occurs during the regenerative phase starting approximately around weeks 4–6 post-operatively [26].

The significant correlation between glutamate levels and PINP observed only in the FWB group suggests that increased glutamate levels, via direct functional weight-bearing, regulates collagen type I synthesis, although PINP at this time point did not display any differences between the two groups. A study by Langberg et al. supports the finding that physical exercise regulates local collagen type I synthesis. They demonstrated that physical training resulted in increased collagen type I synthesis in the peritendinous Achilles tendon [19]. The glutamate concentrations did not only correlate to PINP but also to improved functional outcome at 6 months, confirming that glutamate is vital for healing and suggesting that glutamate may act as a marker for mid-term functional outcome after ATR. Therefore, we suggest further studies on how rehabilitation protocols can upregulate glutamate levels and improve patient functional outcome.

The strengths of the present study include the prospective randomized design with CONSORT methodology. In this study, none of the 56 patients were lost to follow-up. To the best of our knowledge, this is by far the biggest dataset presented in the literature of microdialysis, and especially of the peritendinous space of the Achilles tendon. A limitation to our study is that we have introduced selection bias by introducing 15 patients from a previous study; however, the microdialysis results from the two cohorts did not differ significantly. In addition, the amount of movement in the cast until 2 weeks post-operative in the IMM group is unknown. Although the IMM group did not weight-bear for 2 weeks post-operative, there is a possibility that some range of movement was possible and this may influence the healing process.

From a clinical perspective, this study supports early functional weight-bearing rehabilitation after Achilles tendon repair. It improves the early healing response, and early ankle range of motion is improved without the risk of Achilles tendon elongation.

## Conclusion

In conclusion, this prospective randomized controlled trial demonstrated that direct post-operative weight-bearing and functional mobilization resulted in an early improved healing response compared to non-weight-bearing and immobilization. Weight-bearing resulted in an upregulation of glutamate, which reveal an important mechanism of early tendon healing. Glutamate may prove to become a marker for collagen I synthesis and improved mid-term functional outcome.

## Electronic supplementary material

Below is the link to the electronic supplementary material.

Supplementary material 1 (DOC 219 kb)

## Abbreviations

AT: Achilles tendon
ATR: Achilles tendon rupture
CONSORT: Consolidated standards of reporting trials
FWB: Functional weight-bearing mobilization
LSI: Limb symmetry index
IMM: Non-weight-bearing plaster cast immobilization
PINP: Procollagen I N-terminal propeptide
PIIINP: Procollagen III N-terminal propeptide
RCT: Randomized controlled trial
